# Hyperbaric oxygen therapy increases telomere length and decreases immunosenescence in isolated blood cells: a prospective trial

**DOI:** 10.18632/aging.202188

**Published:** 2020-11-18

**Authors:** Yafit Hachmo, Amir Hadanny, Ramzia Abu Hamed, Malka Daniel-Kotovsky, Merav Catalogna, Gregory Fishlev, Erez Lang, Nir Polak, Keren Doenyas, Mony Friedman, Yonatan Zemel, Yair Bechor, Shai Efrati

**Affiliations:** 1 Research and Development Unit, Shamir Medical Center, Zerifin, Israel; 2 The Sagol Center for Hyperbaric Medicine and Research, Shamir (Assaf-Harofeh) Medical Center, Zerifin, Israel; 3 Sackler School of Medicine, Tel-Aviv University, Tel-Aviv, Israel; 4 Bar Ilan University, Ramat-Gan, Israel; 5 Sagol School of Neuroscience, Tel-Aviv University, Tel-Aviv, Israel

**Keywords:** telomere, senescence, aging, hyperbaric oxygen, length

## Abstract

Introduction: Aging is characterized by the progressive loss of physiological capacity. At the cellular level, two key hallmarks of the aging process include telomere length (TL) shortening and cellular senescence. Repeated intermittent hyperoxic exposures, using certain hyperbaric oxygen therapy (HBOT) protocols, can induce regenerative effects which normally occur during hypoxia. The aim of the current study was to evaluate whether HBOT affects TL and senescent cell concentrations in a normal, non-pathological, aging adult population.

Methods: Thirty-five healthy independently living adults, aged 64 and older, were enrolled to receive 60 daily HBOT exposures. Whole blood samples were collected at baseline, at the 30^th^ and 60^th^ session, and 1-2 weeks following the last HBOT session. Peripheral blood mononuclear cells (PBMCs) telomeres length and senescence were assessed.

Results: Telomeres length of T helper, T cytotoxic, natural killer and B cells increased significantly by over 20% following HBOT. The most significant change was noticed in B cells which increased at the 30^th^ session, 60^th^ session and post HBOT by 25.68%±40.42 (p=0.007), 29.39%±23.39 (p=0.0001) and 37.63%±52.73 (p=0.007), respectively.

There was a significant decrease in the number of senescent T helpers by -37.30%±33.04 post-HBOT (P<0.0001). T-cytotoxic senescent cell percentages decreased significantly by -10.96%±12.59 (p=0.0004) post-HBOT.

In conclusion, the study indicates that HBOT may induce significant senolytic effects including significantly increasing telomere length and clearance of senescent cells in the aging populations.

## INTRODUCTION

Aging can be characterized by the progressive loss of physiological integrity, resulting in impaired functions and susceptibility for diseases and death. This biological deterioration is considered a major risk factor for cancer, cardiovascular diseases, diabetes and Alzheimer’s disease among others. At the cellular level, there are two key hallmarks of the aging process: shortening of telomere length and cellular senescence [1].

Telomeres are tandem nucleotide repeats located at the end of the chromosomes which maintain genomic stability. Telomeres shorten during replication (mitosis) due to the inherent inability to fully replicate the end part of the lagging DNA strand [2]. Telomere length (TL), measuring between 4 to 15 kilobases, gradually shorten by ~20-40 bases per year and is associated with different diseases, low physical performance and cortical thinning of the brain [3–5]. When TL reaches a critical length, cells cannot replicate and progress to senescence or programmed cell death [6]. Goglin et al. demonstrated that adults with shorter TLs have increased mortality rates [7]. Shortened TLs can be a direct inherited trait, but several environmental factors have also been associated with shortening TL including stress, lack of physical endurance activity, excess body mass index, smoking, chronic inflammation, vitamins deficiency and oxidative stress [2, 8, 9].

Cellular senescence is an arrest of the cell cycle which can be caused by telomere shortening [10], as well as other aging associated stimuli independent of TL such as non-telomeric DNA damage [1]. The primary purpose of senescence is to prevent propagation of damaged cells by triggering their elimination via the immune system. The accumulation of senescent cells with aging reflects either an increase in the generation of these cells and/or a decrease in their clearance, which in turn aggravates the damage and contributes to aging [1].

A growing body of research has found several pharmacological agents that can reduce the telomere shortening rate [11, 12]. Several lifestyle interventions including endurance training, diets and supplements targeting cell metabolism and oxidative stress have reported relatively small effects (2-5%) on TL^3,^ [2, 8, 9].

Hyperbaric oxygen therapy (HBOT) utilizes 100% oxygen in an environmental pressure higher than one absolute atmospheres (ATA) to enhance the amount of oxygen dissolved in body’s tissues. Repeated intermittent hyperoxic exposures, using certain HBOT protocols, can induce physiological effects which normally occur during hypoxia in a hyperoxic environment, the so called hyperoxic-hypoxic paradox [13–16]. In addition, it was recently demonstrated that HBOT can induce cognitive enhancements in healthy aging adults via mechanisms involving regional changes in cerebral blood flow [17]. On the cellular level, it was demonstrated that HBOT can induce the expression of hypoxia induced factor (HIF), vascular endothelial growth factor (VEGF) and sirtuin (SIRT), stem cell proliferation, mitochondrial biogenesis, angiogenesis and neurogenesis [18]. However, no study to date has examined HBOT’s effects on TL and senescent cell accumulation.

The aim of the current study was to evaluate whether HBOT affects TL and senescence-like T-cells population in aging adults.

## RESULTS

**Figure 1:**
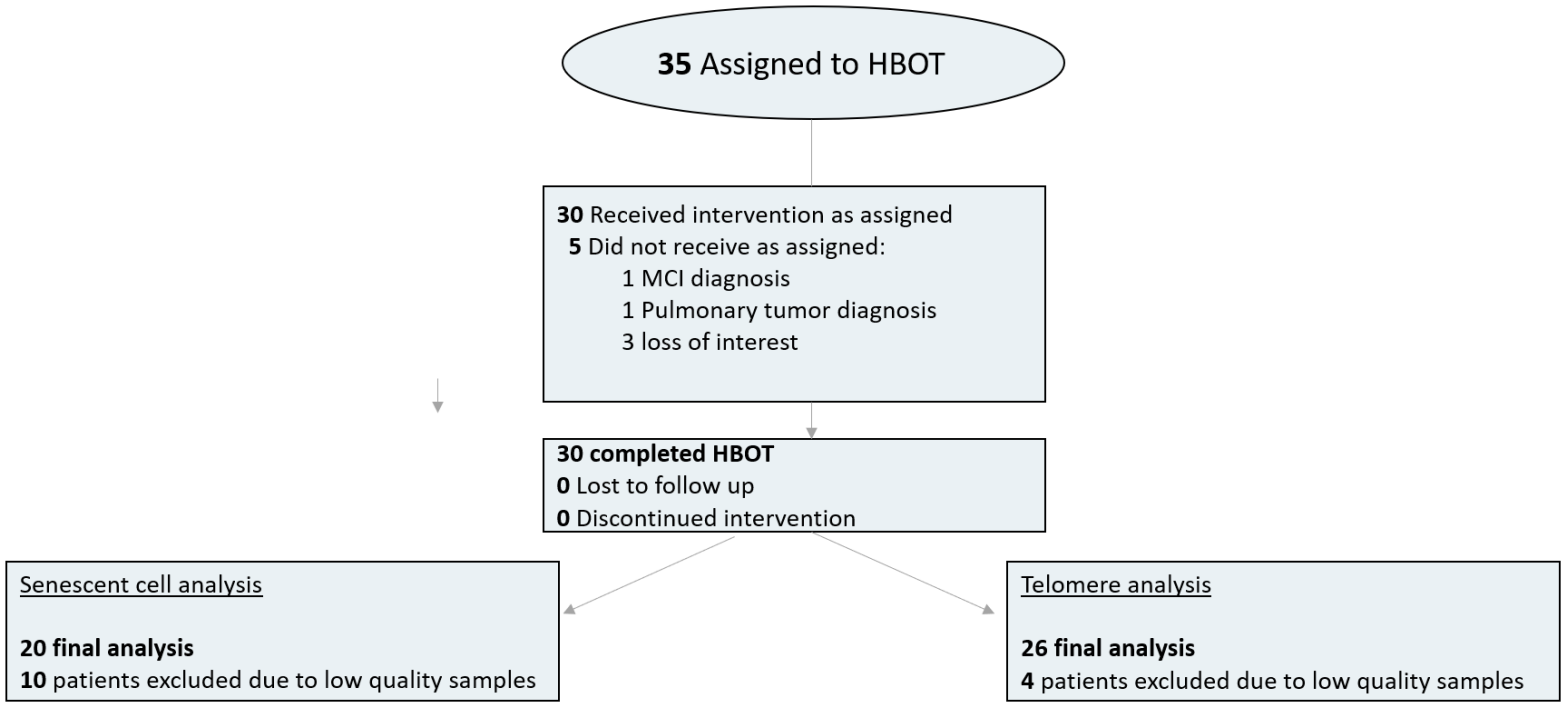
**Patient flowchart.**

Thirty-five individuals were assigned to HBOT. Five patients did not complete baseline assessments and were excluded. All 30 patients who completed baseline evaluations completed the interventions. Due to the low quality of blood samples (low number of cells or technician error), four patients were excluded from the telomere analysis and 10 patients from senescent cell analysis (Figure 1). The baseline characteristics and comparison of the cohorts following exclusion of the patients are provided in Table 1. There were no significant differences between the three groups (Table 1).

**Table 1:** Baseline characteristics.

		**HBOT**	**Telomere analysis**	**Senescent analysis**	**P-value**
**N**		30	25 (83.3%)	20 (66.6%)	
**Age (years)**		68.41±13.2	67.56±14.35	66.70±16.00	0.917
**BMI**		26.77±3.20	26.89±3.34	27.14±3.81	0.946
**Males**		16 (53.3%)	13 (52.0%)	10 (50.0%)	0.987
**Females**		14 (47.7%)	12 (48.0%)	10 (50.0%)	0.987
**Complete blood count**					
	Hemoglobin	6.33±1.25	6.57±1.15	6.58±1.29	0.707
	White blood cells	14.02±1.40	13.92±1.35	13.97±1.49	0.969
	%PBMC	39.96±6.75	39.25±6.64	38.59±6.63	0.774
	Platelets	239.87±1.39	244.08±43.0	254.05±41.4	0.559
**Chronic medical conditions**				
	Atrial fibrillation	4 (13.3%)	4 (16.0%)	2 (10.0%)	0.841
	Hypothyroidism	4 (13.3%)	4 (16.0%)	3 (15.8%)	0.956
	Obstructive sleep apnea	4 (13.3%)	4 (16.0%)	3 (15.0%)	0.961
	Asthma	1 (3.3%)	1 (4.0%)	0	0.680
	BPH	7 (23.3%)	5 (20.0%)	6 (30.0%)	0.733
	GERD	3 (10%)	2 (8.0%)	2 (10.0%)	0.961
	Osteoporosis	5 (16.7%)	5 (20.0%)	4 (20.0%)	0.936
	Rheumatic arthritis	1 (3.3%)	0	1 (5.0%)	0.561
	Osteoarthritis	7 (23.3%)	4 (16.0%)	5 (25.0%)	0.755
	Diabetes mellitus	3 (10%)	3 (12.0%)	2 (10.0%)	0.966
	Hypertension	7 (23.3%)	5 (20.0%)	5 (25.0%)	0.918
	Dyslipidemia	16 (53.3%)	14 (56.0%)	12 (60.0%)	0.897
	Ischemic heart disease	2 (6.7%)	1 (4.0%)	2 (10.0%)	0.725
	History of smoking	10 (33.3%)	8 (32.0%)	7 (35.0%)	0.978
**Chronic medications**				
	Anti-aggregation	8 (26.7%)	6 (24.0%)	5 (25.0%)	0.974
	ACE-Inhibitors/ARB blockers	6 (20%)	6 (24.0%)	6 (30.0%)	0.720
	Beta blockers	5 (16.7%)	5 (20.0%)	3 (15.0%)	0.901
	Calcium blockers	3 (10%)	3 (12.0%)	2 (10.0%)	0.966
	Alpha blockers	7 (23.3%)	5 (20.0%)	6 (30.0%)	0.733
	Diuretics	2 (6.7%)	1 (4.0%)	1 (5.0%)	0.906
	Statins	10 (33.3%)	9 (36.0%)	7 (35.0%)	0.978
	Oral hypoglycemic	1 (3.3%)	1 (4.0%)	1 (5.0%)	0.958
	Bisphosphonates	1 (3.3%)	1 (4.0%)	1 (5.0%)	0.958
	Proton pump inhibitors	3 (10%)	3 (12.0%)	3 (15.0%)	0.726
	Hormones	3 (10%)	3 (12.0%)	2 (10.0%)	0.966
	Benzodiazepines	3 (10%)	2 (8.0%)	1 (5.0%)	0.816
	SSRI	5 (16.7%)	5 (20.0%)	3 (15.0%)	0.990

### Telomere length

**Figure 2:**
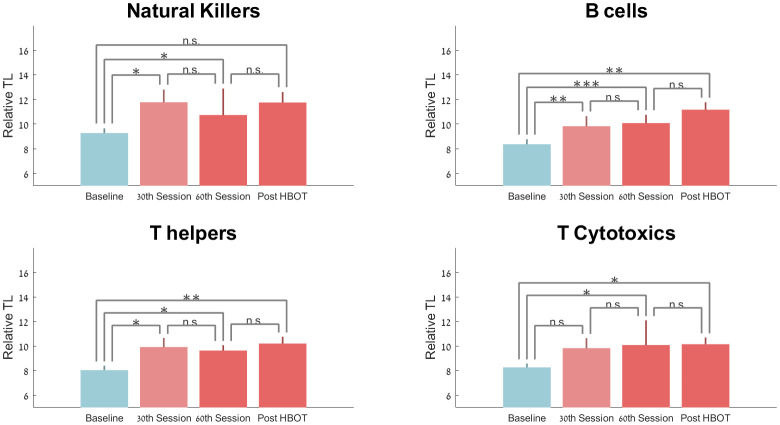
**Telomere length changes with HBOT.** Mean+SEM *p<0.05, **p<0.01, ***p<0.001.

Compared to the baseline, the T-helper telomere lengths were significantly increased at the 30^th^ session and post-HBOT by 21.70±40.05 (p=0.042), 23.69%±39.54 (p=0.012) and 29.30±38.51 (p=0.005), respectively (Figure 2). However, repeated measures analysis shows a non-significant trend (F=4.663, p=0.06, Table 2 and Figure 2).

**Table 2:** Telomere length and senescent cell changes post-HBOT.

		**Absolute changes**				**Relative changes (%)**			**Repeated measures F (p)**
PBMC	**Baseline**	**30^th^ Session**		**60^th^ Session**	**Post HBOT**	**30^th^ session**	**60^th^ session**	**Post-HBOT**	
PBMC ((N=25)	2.55±0.53				-0.15±0.40			-4.91±16.70	1.987 (t) 0.09
PBMC (N=20)	2.50±0.53				-0.13±0.31			-4.21±11.99	1.810 (t) 0.07
**Relative telomeres length (N=25)**								
Natural killer	9.27±1.91	11.77±5.14 (**0.045**)		10.73±2.73 (**0.013**)	11.75±4.22 (0.06)	25.02±51.42	20.56±33.35	22.16±44.81	0.812 (0.391)
B-cells	8.36±2.02	10.22±3.04 (**0.007**)		11.23±3.58 (**0.0001**)	11.17±2.98 (**0.007**)	25.68±40.42	29.39±23.39	37.63±52.73	**7.390 (0.017)**
T Helper	8.04±1.82	9.92±3.68 (**0.042**)		9.63±2.17 (**0.012**)	10.20±2.77 (**0.005**)	21.70±40.05	23.69±39.54	29.30±38.51	4.663 (0.063)
T Cytotoxic	8.26±1.54	9.83±4.08 (0.11)		10.08±3.33 (**0.019**)	10.15±2.74 (**0.023**)	18.29±45.62	24.13±40.88	19.59±33.98	1.159 (0.310)
**Senescent cells (% of T cells) (N=20)**								
T Helper	10.29±5.42	7.84±7.09 (0.09)		8.51±7.45 (0.20)	6.22±4.88 (<**0.0001**)	-19.66±80.03	-11.67±94.30	-37.30±33.04	**8.548 (0.01)**
T Cytotoxic	52.19±21.07	45.53±19.91 (<**0.0001**)		45.45±18.81 (**0.002**)	46.59±21.91 (**0.0004**)	-12.21±8.74	-9.81±9.50	-10.96±12.59	**6.916 (0.018)**

P-values shown in () compared to baseline.

P-values in bold <0.05.

Compared to baseline, telomere lengths of B cells increased significantly at the 30^th^ session, 60^th^ session and post-HBOT by 25.68%±40.42 (p=0.007), 29.39%±23.39 (p=0.0001) and 37.63%±52.73 (p=0.007), respectively (Figure 2). Repeated measures analysis shows a significant within-group effect (F=0.390, p=0.017, Table 2 and Figure 2).

Compared to baseline, natural killer cells telomer lengths significantly increased at the 30^th^ session (p=0.045) and at the 60^th^ session by 20.56% ±33.35 (p=0.013). Post-HBOT, telomere lengths increased by 22.16%±44.81 post-HBOT (p=0.06, Table 2 and Figure 2). Repeated measures analysis indicates that there was no additional significant effect after the 30^th^ session (F=0.812, p=0.391).

Compared to baseline, cytotoxic T-cells had a non-significant increase at the 30^th^ session by 18.29%±45.62 (p=0.11), followed by a significant increase of 24.13%±40.88 at the 60^th^ session (p=0.0019) and 19.59%±33.98 post-HBOT (p=0.023). Repeated measures analysis indicates that there was no additional significant effect after the 30^th^ session (F=1.159, p=0.310, Table 2 and Figure 2).

### Senescent cells

**Figure 3:**
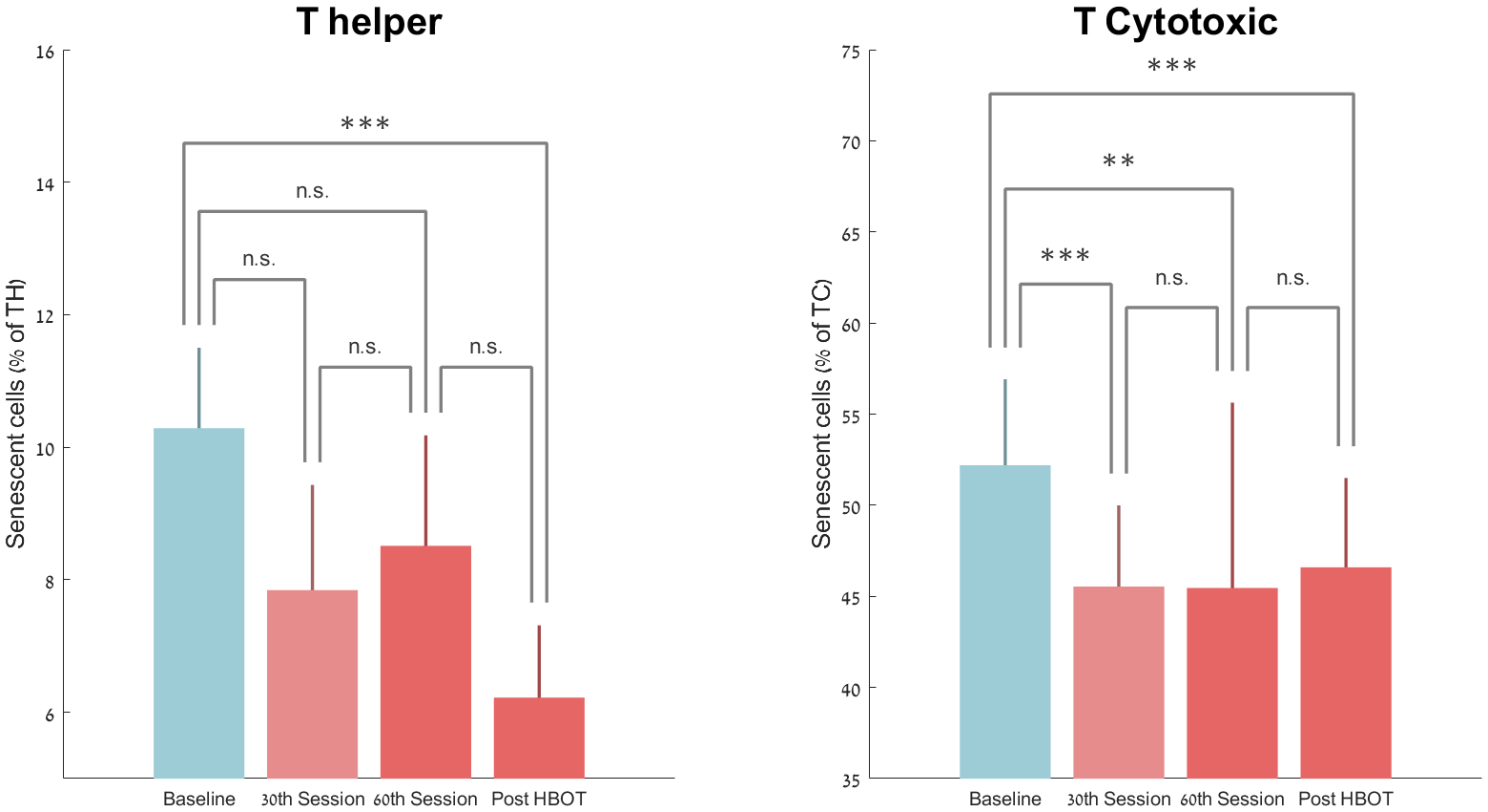
**Senescent cell changes with HBOT.** Mean+SEM *p<0.05, **p<0.01, ***p<0.001.

There was a non-significant decrease in the number of senescent T-helpers at the 30^th^ session and 60^th^ session by -19.66%±80.03 (p=0.09) and -11.67%±94.30 (p=0.20) respectively. However, there was a significant drop in the number of senescent T helpers by -37.30%±33.04 post-HBOT (P<0.0001, Figure 3). Repeated measures analysis showed a significant continuous effect even after the 30^th^ session, with a within-group effect (F=8.547, p=0.01, Table 2 and Figure 3).

T-cytotoxic senescent cell percentages decreased significantly by -12.21%±8.74 (P<0.0001) at the 30^th^ HBOT session, -9.81%±9.50 at the 60^th^ HBOT session (0.002) and -10.96%±12.59 (p=0.0004) post-HBOT (Table 2 and Figure 3). Repeated measures analysis shows a significant continuous effect even after the 30^th^ session, with a within-group effect (F=6.916, p=0.018, Table 2).

### HIF-1alpha

HIF-1alpha levels were increased from 10.54±3.39 to 19.71±3.39 at the 60^th^ session (p=0.006) where 2 weeks post HBOT levels of 16.81±7.65 were not significantly different from baseline (p=0.16).

## DISCUSSION

In this study, for the first time in humans, it was found that repeated daily HBOT sessions can increase PBMC telomere length by more than 20% in an aging population, with B cells having the most striking change. In addition, HBOT decreased the number of senescent cells by 10-37%, with T helper senescent cells being the most effected.

A substantial number of associations between telomere length and lifestyle modifications have been observed. This has led to several interventional studies which included diet, supplements (such as omega-3, and walnuts among others), physical activity, stress management and social support. A two year trial conducted on cognitively healthy elderly adults, using a diet rich in walnuts, showed a non-significant trend to preserve telomere length when compared to a control diet [19]. In another study which evaluated the effect of a twelve week low frequency explosive-type resistance training in elderly people, telomere length was better preserved in the intervention group without a significant increase [20]. A recent study found that aerobic endurance training or high intensity interval training for six month increased telomere length up to 5% [21]. Additional weight loss, yoga and stress management techniques failed to show significant telomere length changes [22–25]. However, most of these studies have shown significant correlations between antioxidant activity and telomerase activity [22–25].

While many genetic and environmental factors are associated with telomere shortening, the most common suggest mechanism is oxidative stress. Oxidative stress can occur from imbalances between the production of reactive oxygen species (ROS) and cellular scavengers. Telomeres are highly sensitive to oxidative DNA damage, which can induce telomere shortening and dysfunction [26]. The association between oxygen and/or oxidative stress and telomere length has been debated for the past several decades. Human cell culture studies consistently show that mild oxidative stress accelerates telomere shortening, whereas antioxidants and free radical scavengers decrease shortening rates and increase the cellular proliferative lifespan [27]. Several clinical studies on pathological conditions (such as diabetes, inflammatory diseases, Parkinson’s disease) have shown correlations between oxidative stress markers, reactive oxygen species scavengers levels and telomere length [28]. However, healthy individuals did not show similar results [29].

Exposing cell cultures to a hyperbaric environment has been previously suggested to induce significant oxidative stress and premature cells senescence [30]. However, this was based on isolated cells grown in a hyperbaric incubator and not on the complex biological system of humans as in this study. Similar to the current study, a previous prospective one-year observational study in divers exposed to intense hyperbaric oxygen, showed significant telomere elongation in leukocytes [31]. As used in the current study, the HBOT protocol utilizes the effects induced by repeated intermittent hyperoxic exposures, the so called hyperoxic hypoxic paradox [13, 18]. These intermittent hyperoxic exposures induce an adaptive response which includes increased upregulation of antioxidants genes [32] and production of antioxidants/scavengers that adjust to the increased ROS generation causing the ROS/scavenger ratio to gradually becomes similar to the ratio under a normal oxygen environment. However, because the scavenger elimination half-life (T_1/2_) is significantly longer than the T_1/2_ of ROS, upon return to normoxia, following repeated hyperoxic exposures, there are significantly higher levels of scavengers and increased antioxidant activity [13, 18]. Thus, similar to physical exercise and caloric restriction, a daily repeated HBOT protocol can induce the hormesis phenomenon. Single exposures increase ROS generation acutely, triggering the antioxidant response, and with repeated exposures, the response becomes protective [13, 18].

Additionally, intermittent hyperoxic exposures induce many of the physiological responses that occur during hypoxia [13]. HBOT induces the release of transcription factors called hypoxic induced factors (HIF) and increase their stability and activity [14]. In turn, HIF induces a cellular cascade including vascular endothelial growth factor and angiogenesis induction, mitochondria biogenesis, stem cells mobilization and SIRT1 increased activity [18]. Our study confirms increased HIF expression is induced by repetitive HBOT exposures, which gradually decreases towards normalization of HIF levels at nonmonic environment.

Currently, many interventions that genetically or pharmacologically (senolytic drugs) remove senescent cells have been developed in animal models and are waiting for safety and efficacy evaluations in humans [33]. The current study suggests a non-pharmacological method, clinically available with well-established safety profile, for senescent cells populations decrease. Our protocol included 60 sessions of 100% oxygen at 2 ATA including three air breaks during each session to utilize the hyperoxic hypoxic paradox and minimize the risk of oxygen toxicity. Interestingly, both TL and senescent cell reduction peaked at the 30^th^ session. However, the dose response curve related to the applied pressure, time and number of HBOT exposures and its relation to HIF expression and its related regenerative effects are still not fully understood and further studies are needed to find the optimal HBOT protocols.

Hyperbaric oxygen therapy is a well-established treatment modality for non-healing wounds, radiation injuries as well as different hypoxic or ischemic events (such as carbon monoxide toxicity, infections, etc). In recent years, a growing evidence from pre-clinical as well as clinical trials demonstrate the efficacy of HBOT for neurological indications including idiopathic sudden sensorineural hearing loss [34], post stroke and post traumatic brain injury [35–41], central sensitization syndrome such as fibromyalgia syndrome [42, 43] and age related cognitive decline [17] and animal models of Alzheimer’s disease [44]. For the first time, the current study aimed to evaluate the physiological effect on the cellular level in aging humans without any functional limiting disease.

### Study limitations

The current study has several limitations and strengths to consider. First, the limited sample size has to be taken into account. Second, the lack of control group. However, the study suggests impressive results on TL and senescent cell clearance, which weren't observed in other interventions. Moreover, the baseline telomere length values of our cohort match the expected values for the aging population [45–47]. Third, the duration of the effect has yet to be determined in long-term follow-ups. Fourth, telomerase activity was not evaluated due to the method chosen for blood preservation and evaluation. Nevertheless, several strengths should be stressed. In this study, CD28 was used as a biomarker for senescent cells whereas CD57 was not available as a confirmatory marker for T cell senescence. Biomarkers were assessed on specific leukocytes populations rather than using the entire PBMCs as one group. The isolated HBOT effect was measured and participants were monitored for not making any lifestyle changes (such as nutrition and exercise), medications or any other intervention that may have acted as possible confounders.

In summary, the study indicates that HBOT can induce significant senolytic effects, including significant increased telomere length and clearance of senescent cells in aging populations.

## MATERIALS AND METHODS

### Subjects

Thirty-five adults without pathological cognitive declines, aged 64 and older, who lived independently in good functional and cognitive status, were enrolled. The study was performed between 2016-2020 in the Shamir (Assaf-Harofeh) Medical Center, Israel. Included patients did not have cardiac or cerebrovascular ischemia histories for the last year prior to inclusion. Exclusion criteria included: previous treatment with HBOT for any reason during the last three months, any history of malignancy during the last year, any pathological cognitive decline, severe chronic renal failure (GFR <30), uncontrolled diabetes mellitus (HbA1C>8, fasting glucose>200), immunosuppressants, MRI contraindications (including BMI>35), active smoking or pulmonary diseases.

### Study design

The study protocol was approved by Institutional Review Board of the Shamir Medical Center, Israel. The study was performed as a prospective clinical trial. After signing an informed consent and undergoing a baseline evaluation, the subjects were assigned to HBOT. Measurement points were evaluated at baseline, half-point of the treatment protocol (30^th^ session), the day of the last HBOT session and 1-2 weeks after the HBOT.

The study cohort included only patients treated by HBOT, which is part of a larger cohort of normal ageing population studied at the Shamir medical center, Israel (NCT02790541 [17]).

### Interventions

The HBOT protocol was administrated in a Multiplace Starmed-2700 chamber (HAUX, Germany). The protocol comprised of 60 daily sessions, five sessions per week within a three-month period. Each session included breathing 100% oxygen by mask at 2ATA for 90 minutes with 5-minute air breaks every 20 minutes. Compression/decompression rates were 1 meter/minute. During the trial, neither lifestyle and diet changes, nor medications adjustments were allowed.

### Blood samples

Whole blood samples were collected into EDTA tubes using a standard technique, at baseline, at the half-point of the HBOT protocol (30^th^ session), the day of the last HBOT session (60^th^ session) and 1-2 weeks following the last HBOT session.

### Peripheral blood mononuclear cells (PBMCs) isolation

Whole blood was diluted using phosphate buffered saline (PBS). Density gradient separation was performed using Leucosep tubes filled with Lymphoprep. The tubes were then centrifuged at 1000×*g* for 10 min at 25° C degrees. Following centrifugation, the cell layers (buffy coat) were immediately collected via pipette and transferred to 50 mL conical centrifuge tubes, resuspended with sufficient 1X PBS to a volume of 50 mL and centrifuged at 300×*g* for 10 min at 25° C degrees. Following removal of the supernatant, each sample was labeled.

### Telomere length

**Figure 4:**
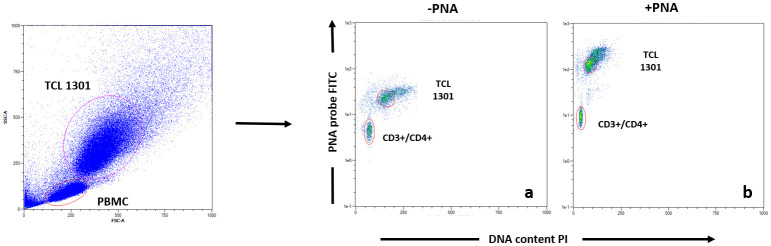
**Example of Flow Fish data analysis of T helper subpopulation.** Each blood sample was either stained with PNA probe (**b**) or without (**a**), following by antibodies staining (CD3, CD4, CD8, CD16, CD19), before data acquisition.

Telomeres were labelled according to the Dako PNA/FITC kit protocol (Code K5327). On a single cell suspension consisting of a mixture of PBMCs (sample cells) and TCL 1301 cell line (control cells), the DNA was denatured for 10 minutes at 82° C in a microcentrifuge tube either in the presence of hybridization solution without probe or in hybridization solution containing the fluorescein-conjugated PNA telomere probe. The hybridization took place in the dark at room temperature (RT) overnight. The hybridization was followed by two 10-minute post-hybridization washes with a wash solution at 40° C. The sample was then labeled with CD4+, CD8+, CD3+, CD19+ and CD56+ conjugated antibodies in an appropriate buffer for further flow cytometric analysis [48, 49]. Each sample was run in duplicate. Following flow cytometric analysis, the relative telomere length (RTL) was calculated for CD3+/CD4+ (T-helper), CD3+/CD8+ (T-cytotoxic), CD3+/CD56+ (natural killer) and CD19+ (B-cells). The RTL value was calculated as the ratio between the telomere signal of each sample and the control cell (TCL 1301 cell line) with correction for the DNA index of G0/1 cells. Sample cells and control cells were analyzed separately for DNA ploidy using propidium iodide staining to standardize the number of telomere ends per cell and thereby telomere length per chromosome. See Figure 4 for FACS analysis example.

### Immunophenotyping

Percentages of CD3+CD4+CD28-null T cells (senescent T helpers) and CD3+CD8+CD28-null T cells (senescent T cytotoxics) were determined by flow-cytometric analysis. PBMC were stained with VioBlue conjugated anti-CD3, Viogreen conjugated anti-CD8, PE-VIO 770A conjugated anti-CD4 and APC-VIO 770A anti-CD28 antibodies (Miltenyi Biotec). Cells were analyzed with a MACSQuant Flow Cytometer (Miltenyi Biotec). The percentage of CD28null T cells within the CD4+ or CD8+ T cell population was then calculated.

### Hypoxia induced factor (HIF-1alpha)

Intracellular HIF1a staining was performed with APC conjugated anti-HIF1a antibody or corresponding Isotype Control (R&D systems) following fixation and permeabilization *(Life Technologies).* Cells were analyzed with a MACSQuant Flow Cytometer (Miltenyi Biotec) and the percentage of HIF1a expressing PBMCs, was determined.

### Statistical analysis

Unless otherwise specified, continuous data were expressed as means ± standard-deviation. The normal distribution for all variables was tested using the Kolmogorov-Smirnov test. One-way ANOVA was performed to compare variables between and within the three groups at baseline.

Categorical data is expressed in numbers and percentages and compared by chi-square tests. Univariate analyses were performed using Chi-Square/Fisher’s exact test to identify significant variables (P<0.05).

To evaluate HBOT’s effects, a repeated measures ANOVA model was used to test the main within-subject effect. Post hoc tests on the means was conducted to test for time differences using t tests with a Bonferroni correction.
